# Supplementary material for: A Cross-Sectional Retrospective Study of Non-Splenectomized and Never-Treated Patients with Type 1 Gaucher Disease
Source: J Clin Med. 2020 Jul 22;9(8):2343. doi: 10.3390/jcm9082343 (PMC7464688; doi:10.3390/jcm9082343)
Supplement: Supplementary file 1 [file jcm-09-02343-s001.pdf]

Supplementary material

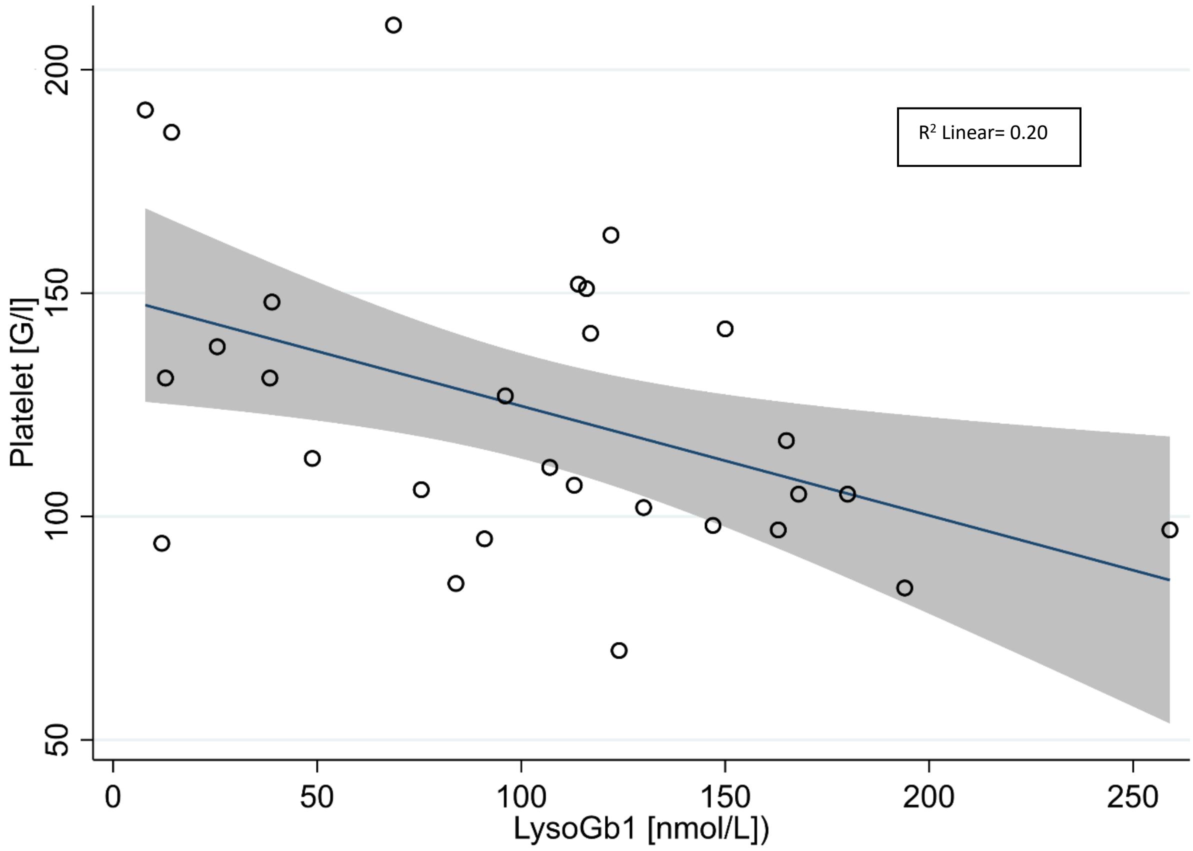

Figure S1. Correlation between lyso-Gb1 and platelet count at the last evaluation.
